# Emotional memory for musical excerpts in young and older adults

**DOI:** 10.3389/fnagi.2015.00023

**Published:** 2015-03-12

**Authors:** Irene Alonso, Delphine Dellacherie, Séverine Samson

**Affiliations:** ^1^Laboratoire PSITEC (EA 4072), Université de LilleVilleneuve d'Ascq, France; ^2^Epilepsy Unit, Hôpital de la Pitié-SalpêtrièreParis, France; ^3^Inserm U 1127, CNRS UMR 7225, Sorbonne Universités, UPMC Univ Paris 06 UMR S 1127, Institut du Cerveau et de la Moelle Épinière - ICM, Centre de Neuroimagerie de Recherche - CENIRParis, France; ^4^Centre National de Référence des Maladies Rares, Service de Neuropédiatrie, CHRU de Lille, Université de Lille 2Lille, France

**Keywords:** emotion, memory, consolidation, valence, arousal, music, aging

## Abstract

The emotions evoked by music can enhance recognition of excerpts. It has been suggested that memory is better for high than for low arousing music (Eschrich et al., 2005; Samson et al., 2009), but it remains unclear whether positively (Eschrich et al., 2008) or negatively valenced music (Aubé et al., 2013; Vieillard and Gilet, 2013) may be better recognized. Moreover, we still know very little about the influence of age on emotional memory for music. To address these issues, we tested emotional memory for music in young and older adults using musical excerpts varying in terms of arousal and valence. Participants completed immediate and 24 h delayed recognition tests. We predicted highly arousing excerpts to be better recognized by both groups in immediate recognition. We hypothesized that arousal may compensate consolidation deficits in aging, thus showing more prominent benefit of high over low arousing stimuli in older than younger adults on delayed recognition. We also hypothesized worst retention of negative excerpts for the older group, resulting in a recognition benefit for positive over negative excerpts specific to older adults. Our results suggest that although older adults had worse recognition than young adults overall, effects of emotion on memory do not seem to be modified by aging. Results on immediate recognition suggest that recognition of low arousing excerpts can be affected by valence, with better memory for positive relative to negative low arousing music. However, 24 h delayed recognition results demonstrate effects of emotion on memory consolidation regardless of age, with a recognition benefit for high arousal and for negatively valenced music. The present study highlights the role of emotion on memory consolidation. Findings are examined in light of the literature on emotional memory for music and for other stimuli. We finally discuss the implication of the present results for potential music interventions in aging and dementia.

## Introduction

It has been well established that emotional events are better memorized than non-emotional events (for a review see LaBar and Cabeza, 2006; Bennion et al., 2013; Talmi, 2013). Listening to music can evoke powerful emotions (Juslin and Västfjäll, 2008; Koelsch, 2010; Cochrane et al., 2013) that can subsequently influence memory. A growing number of studies suggest that music can produce durable memories in normal as well as pathological aging, and that this could be due in part to emotion (Bartlett and Snelus, 1980; Bartlett et al., 1995; Schulkind et al., 1999; Janata et al., 2007; Samson et al., 2009; Simmons-Stern et al., 2010).

To investigate the effects of emotion on memory, researchers have applied emotional memory paradigms, which compare memory for stimuli with different emotional characteristics. Most studies in emotional memory have adopted a dimensional approach that characterizes emotions in a graded continuum space that is defined classically by two main dimensions: arousal and valence (Russell, 1980; Bradley, 1994). On the one hand, arousal provides a representation of excitement and calmness. High-arousing stimuli have generally showed a mnemonic advantage over low-arousing stimuli (for a review, see Dolcos and Denkova, 2014). High arousal has been shown to enhance memory both at the initial encoding of events, by boosting attention, as well as during consolidation (for a review see Hamann, 2001; Mather and Sutherland, 2011). On the other hand, valence refers to the positive or negative emotion of the stimulus. In non-musical domains, both positive and negative stimuli have shown to enhance memory as opposed to non-emotional stimuli (Hamann et al., 1999; Dolcos and Cabeza, 2002). Nevertheless, a number of studies speak to a benefit in recognition of negative events as compared to positive events, for words (Inaba et al., 2005) and pictures (Mickley and Kensinger, 2008) suggesting an effect of valence on memory favoring negative stimuli (for a review, see Kensinger, 2007).

Most importantly, it may become difficult to interpret the results of studies that look only at one dimension without controlling for the other. For example, negative stimuli have been regularly described to be better encoded than neutral stimuli (Inaba et al., 2005; Murty et al., 2009), but it is worth noting that such stimuli may also be more arousing overall (Ortony et al., 1983; Denburg et al., 2003; Feng et al., 2014, for a review see Mather and Sutherland, 2009). Looking at both valence and arousal dimensions, Adelman and Estes (2013) have shown that the memory benefit for emotionally valenced words, whether positive or negative, over neutral words may be independent of arousal. The necessity to test the conjoint effect of valence and arousal is further supported by neuroimaging studies, which suggest that arousal and valence influence memory through two distinct neural mechanisms (Dolcos et al., 2004; Kensinger and Corkin, 2004; for a review see Murty et al., 2010; Dolcos et al., 2012).

However, it has been shown that effects of arousal and valence on memory may interact (Libkuman et al., 2004; Jefferies et al., 2008; Mickley Steinmetz et al., 2010a; Citron et al., 2014). An interaction between valence and arousal has been shown, for instance, in patients with amygdala lesions (Berntson et al., 2007), whose recognition of negative scenes was modulated by arousal levels, without an effect of arousal on the recognition of positive scenes. This combined evidence highlights the importance of accounting for both emotional arousal and valence of the stimuli to properly assess the independence and/or the interaction of these emotional effects (Kensinger, 2008; Mickley Steinmetz et al., 2010a; Adelman and Estes, 2013; Feng et al., 2014; Gallant and Yang, 2014; for a review see Levine and Pizarro, 2004; Libkuman et al., 2004; Kensinger, 2009; Mather and Sutherland, 2009).

Emotion can influence memory both at encoding and during consolidation (Hamann, 2001). First, arousal (Bradley et al., 1992; MacKay et al., 2004; Mickley Steinmetz et al., 2010b) and valence (MacKay et al., 2004; Thomas, 2006; Talmi et al., 2007) may boost attention during encoding, facilitating the initial processing of information, and consequently, enhancing immediate recognition. As it has been previously discussed, high arousal and negative valence may enhance encoding, resulting in better immediate recognition for these stimuli as compared to low arousing and positively valenced stimuli respectively (MacKay et al., 2004; for a review see Kensinger, 2007). Second, emotion can further modulate memory after encoding through consolidation. Memory consolidation consists of the stabilization of new memories, whereby some memory traces are strengthened to be later retained and other memory traces will become weaker and be subsequently forgotten (for a review see McGaugh, 2000). The reorganization process occurring through consolidation requires a delay and is mainly driven by hormonal release (Buchanan and Lovallo, 2001), being particularly boosted by sleep. Effects of emotion on consolidation are therefore captured by recognition tests with longer delays, particularly after 24 h (Wagner et al., 2005, 2006; Hu et al., 2006; Payne and Kensinger, 2011; Bennion et al., 2015). Although both arousal and valence have shown to affect the initial encoding of events, evidence for long-term effects of emotion on memory has traditionally highlighted the influence of arousal (Bradley et al., 1992). Several lines of evidence suggest that arousal can critically modulate consolidation (Kuhlmann and Wolf, 2006; for a review see Hamann, 2001; Mather and Sutherland, 2011), with high arousing stimuli facilitating delayed recognition. Relatively few studies have investigated the role of valence on consolidation showing an enhancement for negatively as compared to positively valenced stimuli after at least 24 h delay (Kensinger et al., 2007; Waring and Kensinger, 2009). However, this remains an open area of research.

In the musical domain, few studies have investigated emotional memory (Eschrich et al., 2005, 2008; Samson et al., 2009; Aubé et al., 2013; Vieillard and Gilet, 2013; Altenmüller et al., 2014). Eschrich et al. (2005) manipulated valence (positive to negative), arousal (very arousing to very pacifying) and emotional strength (very strong emotions to no emotions) of Bach piano music excerpts to address the effects of emotion on memory for music. Memory for the studied excerpts was tested 2 weeks after encoding with a remember/known paradigm. Arousal was shown to be the main dimension affecting recognition, with better memory for very arousing than for very pacifying Bach excerpts. To further explore these effects, another study was conducted (Eschrich et al., 2008) presenting emotional film music excerpts with a relatively long length of 20–30 s. Similarly to the previous study, the authors manipulated valence, arousal and emotional intensity dimensions, although valence ratings only ranged from less positive to very positive, thus excluding the assessment of negative emotions. Participants were split into two groups with different incidental encoding tasks: an emotional rating task promoting deep encoding or a time estimation task promoting more shallow encoding. Then, the next day, stimuli were presented again to reinforce consolidation. On the third day, participants were given a surprise old/new recognition test. No differences were found between the deep and the shallow encoding groups. In contrast with the previous study (Eschrich et al., 2005), the results did not confirm the effect of arousal or of emotional intensity on recognition but revealed an effect of valence on recognition, with better memory for very positive music than for less positive excerpts. However, in a recent neuroimaging study, Altenmüller et al. (2014) failed to replicate this behavioral recognition benefit for very positive over less positive music following the encoding of film excerpts including some of the same excerpts from Eschrich et al. (2008) and few similar new excerpts. Furthermore, the lack of negative excerpts in these two studies limits the interpretation of the results that do not seem to support findings from non-musical domains, which indicate that negative objects may be better recognized than positive objects (for a review see Kensinger, 2007). Thus, although high positivity of musical excerpts seems to enhance recognition, it remains unclear whether high negativity of excerpts could have also provide an advantage for music memory in healthy young adults.

Other studies on emotional memory for music have used computer-generated midi musical excerpts recorded with a piano timbre (Samson et al., 2009; Aubé et al., 2013; Vieillard and Gilet, 2013). These stimuli were created and validated to express happy, fearful, peaceful, or sad emotions (Gosselin et al., 2005; Vieillard et al., 2008). In a first study (Samson et al., 2009), the participants listened to the stimuli four times and recognition was incidentally assessed after 24 h delay. Recognition was higher for happy and fearful than for sad and peaceful excerpts. As pointed out by the authors, better recognition of scary music seems to be attributed to a confounding factor (higher discriminability between fear stimuli as compared to the other types of stimuli). However, successful recognition of happy music suggests efficient consolidation of either high arousing or positively valenced stimuli. More recently Aubé et al. (2013) conducted a series of experiments focusing on the impact of emotion expressed by music on immediate recognition in young adults. By using short segment of music (1.5 s), the authors confirmed better recognition of both happy and fearful excerpts above the other categories indicating an effect of arousal in recognition memory. However, when the number of events was kept constant (instead of the duration of excerpts) recognition memory enhancement was obtained only for the fearful excerpts. The authors interpret these results as an advantage for threat-related processing as compared to the other emotional categories, although the higher discriminability of target and foils of the fear stimuli previously noted may partially explain the memory results.

All in all, although an effect of arousal on delayed recognition has been suggested (Eschrich et al., 2005), subsequent studies have not succeeded to disentangle the effect of arousal from the effect of valence (Samson et al., 2009; Aubé et al., 2013). Furthermore, the effect of valence on recognition remains controversial, being unclear whether negative (Aubé et al., 2013) or positive valence music (Eschrich et al., 2008; Samson et al., 2009) may be better recognized.

Moreover, and of great relevance for the present study, effects of emotional memory may vary with aging (for a review see Mather, 2004). Aging is typically associated with memory decline, and weakened consolidation (Scullin, 2013). However, it seems that older adults are not only generally less efficient than the young, but also use different strategies to retain items and events (Carstensen and Turk-Charles, 1994; Gallant and Yang, 2014; for a review see Mather, 2004). A memory advantage for positive stimuli over neutral or negative stimuli, together with diminished processing of negative stimuli has been frequently reported in aged (Charles et al., 2003; Mather and Carstensen, 2005; Mather and Knight, 2005; Thomas, 2006) as compared to younger adults (for a review see Mather, 2006; Reed et al., 2014). Such an emotional bias in aging has been described for words (Leigland et al., 2004; Kensinger, 2008) (for contrasting results see Grühn et al., 2005), pictures (Charles et al., 2003), as well as for emotional processing in music (Lima and Castro, 2011; Vieillard and Gilet, 2013).

In contrast, few studies suggest that valence effects on consolidation do not differ with aging, either when showing an advantage for negative stimuli as compared to positive stimuli (Comblain et al., 2004; Kensinger et al., 2007; Kensinger and Schacter, 2008; Waring and Kensinger, 2009) or vice versa (Denburg et al., 2003). Regarding the effect of arousal, enhancing recognition of high arousing events has been frequently demonstrated in aging (Kensinger et al., 2002; Otani et al., 2007; Kensinger, 2008), even after a 24 h delay (Waring and Kensinger, 2009). Although general consolidation deficits have been described in older adults (Cherdieu et al., 2014), it is possible that arousal may exert a compensatory role, which may result in greater benefit of high arousing events after a delay for older than for young adults. Such an interpretation has recently been challenged in a study investigating immediate and 24 h delayed memory of emotional pictures in aging (Leal and Yassa, 2014). Results in this study suggest that whereas older adults (as opposed to younger adults) benefit from high arousing pictures for immediate recognition, emotional memories in the older group are dramatically forgotten after 24 h. This discrepancy between findings underlines the need to take immediate and delayed recognition into account, in special for studies comparing young and older adults.

To our knowledge, only one study has addressed the effects of aging on emotional memory for music (Vieillard and Gilet, 2013). This study compared immediate memory recognition for music in young (19–24 years old) and older (60–84 years old) adults using the emotional short computer-generated midi musical excerpts previously described (Gosselin et al., 2005; Vieillard et al., 2008). Although an overall memory advantage was found for young adults as compared to older adults, all participants showed a recognition advantage for fearful excerpts as compared to the other music stimuli, similar to the results reported by Aubé et al. (2013) for young adults. However, memory performance of all participants was very low. This floor effect might have hindered the sensitivity for other possible emotional memory differences. In addition, the lower false alarm rates for the fear stimuli as compared to the other emotional stimuli in this study further reflects the issue of the higher discriminability of fear excerpts (Samson et al., 2009) previously discussed. Differences in the emotional responsiveness to music were also found, whereby older adults showed lower discriminability of happy excerpts together with a decrease in their responsiveness to sad and scary music as compared to young adults.

In the present study we addressed the effect of normal aging on emotional memory, assessing immediate and delayed recognition of musical excerpts by manipulating emotional dimensions, arousal and valence, in young and older adults. First, we predicted a general effect of age on recognition of emotional music, with a reduced recognition performance overall for older adults. Regarding the effect of arousal, we hypothesized a benefit on memory performance for high arousal excerpts in both groups. Furthermore, we expected that arousal would not only benefit immediate recognition but also compensate weakened consolidation in aging. Therefore, we predicted a benefit of high over low arousing stimuli in the delayed recognition test that will be greater for old than for young adults. However, based on previous studies on aging and emotional memory in other domains, we expected an interaction effect of age and valence to be manifested by a bias toward worst recognition of negative excerpts in the older adult group but not in the young group.

## Materials and methods

### Participants

Thirty native French speakers were included in this study. Two different groups were conformed according to age, a group of 15 young adults (mean age = 22 ± 1.8, ranging from 18 to 25 years; 8 female; years of education = 14.2 ± 2.11, ranging from 9 to 16 years) and another group of 15 older adults (mean age = 75.27 ± 8.54, ranging from 63 to 92 years; 10 female; years of education = 12 ± 4.42, ranging from 6 to 20 years). All participants were right handed as determined by the Edinburgh Inventory (Oldfield, 1971). Existence of psychiatric or neurologic conditions, as well as the current intake of any treatment affecting memory was taken as exclusion criteria for both young and old adults. Only participants with scores within the normal range on the Profile of Mood State (POMS) test for anxiety, depression and tiredness, were included in the sample to ensure that none of the participants had mood disorders that could interfere with emotional processing. Both young (mean ± SD = 4.47 ± 2.17) and older (mean ± SD = 3.80 ± 1.61) participants were non-musicians as attested by the Music Expertise Test (Ehrlé, 1998). All older adults had Mini-Mental Status Examination (Folstein et al., 1975) scores greater than 28 (mean ± SD = 29.27 ± 0.88), ensuring that their cognitive function was normal. The two groups did not differed on their level of education (*t* = 1.74; *df* = 20.06; *p* = 0.097). All participants have signed informed consent and the study was carried out following the declaration of Helsinki principles.

### Stimuli

Musical stimuli consisted of 48 symphonic excerpts of 5 s. duration (± 1 s fade in fade out) taken from different symphonies written by composers between 1830 and 1954. We selected this period for its particular emphasis in emotional expression, and chose to use existent symphonies to provide more naturalistic stimuli. However, to control the effect of familiarity, we excluded the most famous composers and symphonies of this period.

We used a 2 by 2 design where two dimensions, valence (positive or negative) and arousal (high or low), were crossed creating 4 different emotional combinations of valence and arousal: positive and high arousing (V+A+), positive and low arousing (V+A−), negative and high arousing (V−A+) and negative and low arousing (V−A−). Four sets of excerpts were selected for each emotional combination (making 16 sets of excerpts) based on emotional agreement ratings in an independent pilot study. In this pilot study, 232 excerpts were rated in terms of valence (positive, negative), and arousal (high, low). One hundred seventy five participants were asked to rate a pseudo-randomized subset of 33 out of the 232 excerpts in terms of valence (positive, negative) and arousal (high, low). Excerpts with the higher percentage of agreement on combined arousal and valence ratings were selected for the present experiment. Overall agreement on the selected sample was 90.01% ± 8.50 for valence, 93.37% ± 8.29 for arousal and 84.94% ± 11.14 for the combination of both.

The 48 stimuli were grouped into 16 sets (4 for each emotional combination) of three related excerpts consisting of one target and two distractors used for immediate and delayed memory conditions. Length and style of each melody were matched to keep certain homogeneity between the target and the comparison excerpts. For this purpose, each set of three excerpts was composed by the same composer to increase homogeneity, except for one set from each emotional combination made with excerpts from different composers. List of symphonies is reported in Table 1 of Supplementary Material. Soloist passages were excluded to limit salient features that could play a role in encoding. All excerpts were normalized to maximal amplitude of 1.2 db. These stimuli were unknown to all participants as confirmed by post-test debriefing.

### Procedure

The experimental task was run on a PC using Psychopy software. The experimental protocol included three phases: encoding, immediate recognition and delayed recognition. Prior to the beginning of the experiment, participants performed training during which they were shown an example of the test, ensuring they understood the task. During the encoding phase, subjects were presented with an intentional deep encoding task, being instructed to rate valence (positive/negative) and arousal (high/low) of 16 items and to retain them for a recognition test. The order of presentation of arousal and valence scales was counterbalanced across subjects. To reinforce encoding, subjects had an additional presentation of stimuli without the emotional rating task. Immediately after encoding, an old/new recognition test was presented. Sixteen targets were intermixed with 16 distractors in a randomized order. After each musical excerpt, participants had to decide whether or not the excerpt was already heard and to report if they were confident in their response (sure/unsure). After 24 h delay, participants were presented with the delayed recognition test including the 16 targets intermixed with 16 new distractors in a randomized order. Participants were again asked to perform recognition on the stimuli and to provide confidence ratings of their responses.

### Data analysis

Based on the accuracy of the response and the confidence ratings, receiver operating characteristic (ROC) curves were calculated for each arousal and valence combination. Three within-subjects factors were entered in a repeated measures analysis of variance (ANOVA): arousal (high/low), valence (positive/negative), time of testing (immediate/delayed), together with age (young/older) as between-subjects factor.

## Results

### Emotional ratings

Preliminary analyses were carried out to test the effect of age on emotional judgment ratings for arousal and valence of musical excerpts (see Table 1). Ratings of each stimulus were transformed to z-scores and analyzed in two independent analyses of variance for arousal and valence respectively. An ANOVA with one within-subject factor (arousal) and one between-subject factor (age) was carried out on the arousal ratings obtained by young and older adults. The results revealed a main effect of arousal [*F*_(1, 58)_ = 247.59 *p* < 0.001; η^2^ = 0.88], the mean rating being higher for high than for low arousal changes as expected. There was no effect of age, nor any interaction between these factors.

**Table 1:** **Emotional judgement ratings *(z scores)***.

	**Mean rating (mean ± SD)**		
	**Young**	**Aged**	**All**
High arousal	0.64 ± 0.30	0.62 ± 0.43	0.63 ± 37
Low arousal	−0.64 ± 0.42	−0.62 ± 0.41	−0.63 ± 41
Positive valence	0.69 ± 0.32	0.35 ± 0.38	0.52 ± 0.39
Negative valence	−0.69 ± 0.49	−0.35 ± 0.65	−0.52 ± 0.59

Another ANOVA with one within subjects-factor (valence) and one between-subject factor (age) was carried out on the valence ratings by young and older adults. The results show a main effect of valence [*F*_(1, 58)_ = 154.61; *p* < 0.001; η^2^ = 0.73], together with a valence by age interaction [*F*_(1, 58)_ = 6.12; *p* < 0.001; η^2^ = 0.22]. Paired t-tests on the interaction pointed out that older adults rated positive excerpts as being less positive [*t*_(29)_ = 3.74; *p* < 0.05] and negative excerpts less negative [*t*_(29)_ = −2.51; *p* < 0.05] than young adults.

### Recognition

**Figure 1:**
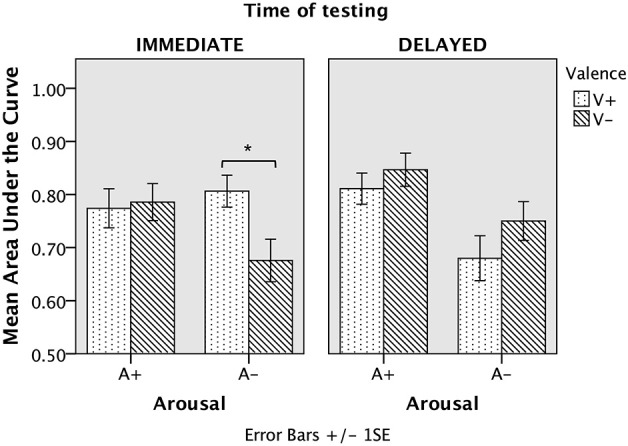
**Mean areas under the ROC curve for all participants from immediate and delayed recognition as a function of arousal and valence**. Origin is set at chance level (0.50). The error bars correspond to the standard error of the mean. ^*^p < 0.0125.

The ANOVA with three within-subjects factors (Valence, Arousal, Time of testing) and one between-subjects factor (Age) was carried out on the areas under the ROC curves. This analysis revealed a main effect of Age [*F*_(1, 28)_ = 26.71; *p* < 0.001; η^2^ = 0.49], the mean area under the curve being lower in old (mean ± SD = 0.74 ± 0.21) than in young adults (mean ± SD = 0.87 ± 0.13). However, the Age factor did not interact with any other factor. A main effect of Arousal was also obtained [*F*_(1, 28)_ = 9.41; *p* < 0.01; η^2^ = 0.25], the mean area under the curve being higher for high (mean ± SD = 0.80 ± 0.18) than for low-arousing excerpts (mean ± SD = 0.73 ± 0.21) in the two groups. There was no main effect of Valence [*F*_(1, 28)_ = 0.02; *p* > 0.05] or of Time of testing [*F*_(1, 28)_ = 0.35; *p* > 0.05], but the analysis revealed an interaction between valence and time of testing [*F*_(1, 28)_ = 11.51; *p* < 0.01; η^2^ = 0.29]. The analysis also showed a Time of testing by Arousal by Valence interaction [*F*_(1, 28)_ = 5.85; *p* < 0.05; η^2^ = 0.17] such that Arousal and Valence differently interacted with scores obtained in immediate and in delayed recognition, as depicted in Figure 1. Thus, we conducted a new analysis including all participants separately for each time of testing to explore arousal by valence interactions.

Immediate recognition results revealed an interaction between Arousal and Valence [*F*_(1, 29)_ = 5.67; *p* < 0.05; η^2^ = 0.16]. To reveal the sense of the interaction, simple effects of Valence on each Arousal level were explored using paired *t*-tests. We used Bonferroni correction of the statistical significance threshold, which was therefore set to *p* < 0.0125 (0.05/4) to correct for multiple comparisons. Whereas we found no difference between positive and negative excerpts for high arousing music, we showed that positive excerpts were better recognized than negative excerpts for low arousing music [*t*_(29)_ = 2.77; *p* < 0.0125]. Simple effects of Arousal on each Valence level did not yield any significant difference (*p* > 0.0125).

Delayed recognition results showed a main effect of Arousal [*F*_(1, 29)_ = 13.92; *p* = 0.001; η^2^ = 0.32], with better recognition of high arousing (mean AUC = 0.83; *SD* = 0.17) than low arousing (mean AUC = 0.71; *SD* = 0.22) excerpts. There was also a main effect of Valence [*F*_(1, 29)_ = 4.83; *p* < 0.05; η^2^ = 0.14] such that negative excerpts (mean AUC = 0.80; *SD* = 0.19) were better recognized than positive excerpts (mean AUC = 0.74; *SD* = 0.20). However, there was no interaction between these two factors (*p* > 0.05).

## Discussion

In this study we have examined the effects of emotion in the recognition of symphonic musical excerpts for young and older adults. Arousal and valence of the musical excerpts were manipulated to address the impact of emotion on memory. Recognition memory tests were administered immediately after the study session, and also after a 24 h delay to measure effects that could be driven by consolidation. Although we confirmed an overall advantage in recognition performance for young over older adults, we were not able to demonstrate any further interaction with age for any of the emotional dimensions in immediate and delayed recognition.

This lack of interaction between age and emotional memory is in line with other studies using different kinds of stimuli such as pictures (Kensinger et al., 2002, 2007; Denburg et al., 2003; Kensinger and Schacter, 2008; Waring and Kensinger, 2009), words (Kensinger et al., 2002; Grühn et al., 2005; Mickley Steinmetz et al., 2010b), scenes (Otani et al., 2007; Murty et al., 2009), and music (Vieillard and Gilet, 2013), which have also failed to show an effect of aging on emotional memory despite the positive bias described in the literature. Mather (2006) suggests that the positive bias effect shown in aging may need strategic and goal-oriented processing of the emotional stimuli to affect memory (see also Mather and Knight, 2005), thus being susceptible to elaboration on the stimuli processing and to participant's motivation. However, as suggested by Dellacherie et al. (2011), real musical performances may intensify motivational significance of the stimuli more than prototypical clips (Vieillard et al., 2008), and thus our stimuli should have successfully engaged participants on the encoding task. Nevertheless, it is possible, that such successful engagement on the task may have minimized potential differences between young and older adults. Importantly, to enhance homogeneity between the different valence/arousal combinations, we limited the music genre to classical music, which might be less emotionally evocative to some participants than other more popular music genres. Thus, further studies sampling a wider range of musical genres are needed to replicate the present emotional memory findings.

Since no interaction with age was found, subsequent assessment of emotional memory was performed for all participants without an age group distinction. We found a double interaction between valence, arousal and time of testing, indicating that arousal and valence had different memory effects at each testing delay. This result supports the idea that early effects of emotion on memory may vary with delay, suggesting that emotions may influence memory consolidation (Talmi, 2013; Leal and Yassa, 2014).

The immediate recognition analysis revealed an interaction between arousal and valence. Whereas valence does not seem to affect recognition of high arousing excerpts, it modifies recognition of low arousing stimuli such that positive excerpts were better recognized than negative excerpts. This result highlights the relevance of valence for low arousing excerpts in immediate testing, in line with Streubel and Kunzmann (2011), who suggest that the positivity bias on emotional reaction may be restricted to low arousing stimuli. Moreover, our finding is in agreement with Kensinger (2008), who also found an interaction whereby more positive non-arousing words were remembered relative to negative non-arousing words for older adults, but no valence effect was found for high arousing words. Nevertheless, an age difference was found in that study, with younger adults having better memory for negative than positive non-arousing words, therefore presenting an effect of valence on low-arousing words in the opposite direction to the older group. Our results also contrast with Leal and Yassa (2014), who found a benefit for emotional valenced pictures over neutral pictures in older adults and the reverse pattern in younger adults. It may be possible that the difference between these previous studies and the present study may be material specific. Most importantly, no particular advantage was found for high arousing negative valence excerpts. As we have previously discussed, few studies have suggested a benefit for “fear” music (Aubé et al., 2013; Vieillard and Gilet, 2013), but such memory effect might have been biased by a difference in the discriminability between the “fear” music and the other emotional music (Samson et al., 2009). Conversely, our results suggest that memory for high arousing excerpts may not differ for negative and positive valence, with only worst memory for negative low arousing excerpts as compared to positive low arousing excerpts.

After a 24 h delay, all participants had better memory for high than low arousing excerpts. The lack of difference between young and older adults on such arousal effect on memory has also been reported for words (Kensinger, 2008) and visual scenes (Otani et al., 2007). The benefit for high arousing music as compared to low arousing music on delayed recognition is in line with Eschrich et al. (2005). This finding highlights the stronger effects of arousal after a delay, supporting the influence of arousal in consolidation. High arousing music remained well remembered also in older adults, in accordance with results from Waring and Kensinger (2009) using emotional images (for opposing results see Leal and Yassa, 2014).

Furthermore, after a 24 h delay, all participants have better memory for negative than positive excerpts. This is in agreement with studies in other domains that have shown a memory advantage for negative over positive stimuli (Waring and Kensinger, 2009). Conversely, our findings are at odds with Eschrich et al. (2008) who found a benefit for very positive musical excerpts over less positive musical excerpts on recognition following a 24 h delay. However, a recent study using similar music stimuli to that of Eschrich et al. (2008) but with a shorter presentation (10 s), has failed to replicate this valence effect on recognition (Altenmüller et al., 2014). It is possible that the advantage for very positive over less positive excerpts may be tied to longer presentations (20–30 s). We cannot exclude, nonetheless, that higher degree of positivity within the positive excerpts, (which is not measured in the present study), may enhance recognition even when negative excerpts could be better recognized. In sum, the valence effect found after 24 h delay, and not during immediate recognition, argues in favor of an enhancement effect of consolidation not only for high arousing stimuli but also for negatively valenced stimuli in line with Waring and Kensinger (2009). Nevertheless, the greater effect size of arousal supports the predominant influence of this dimension on consolidation reported in the literature (for a review see McGaugh, 2000, 2004). Of note, besides the emotional memory immediate and delayed effects, overall memory performance did not seem to decay after 24 h delay, supporting the durability of memory for emotional stimuli. Additionally, despite the interaction between time of test, arousal and valence found in the initial analysis, the presence of a main effect of arousal in that analysis further suggests the stronger impact of this dimension on memory performance overall.

The ability to evoke comparable ratings of high/low arousing and positive/negative valence of the musical excerpts represent a strength of this study. The presence of interactions between arousal and valence on immediate recognition further highlights the pertinence of manipulating emotional dimensions on experiments addressing emotion effects on memory, as it has been already suggested for words (Citron et al., 2014). The analysis of participant's judgment ratings of the music excerpts validated that the emotional characteristics of stimuli regarding arousal and valence were well distinguished. The analysis of the arousal emotional judgment confirmed that arousal ratings correctly reflected arousal differences in the stimuli. Although it has been reported that age can affect the arousal rating of music (Vieillard et al., 2012), no different ratings for arousal were found between age groups for the present stimuli set. Conversely, the analysis on valence ratings showed an interaction between valence and aging, suggesting that older adults rated positive excerpts less positive and negative excerpts less negative than young adults. Differences between older and younger adult groups in the valence of emotional judgments have been already reported for music (Lima and Castro, 2011), indicating more positive ratings for negative stimuli, which can partially account for the present interaction found between ratings of valence and age. Although the valence rating analysis suggests that older adults manipulated a smaller valence range than the young group to judge valence, still positive excerpts were rated more positive than negative excerpts within this group, indicating that they could distinguish valence well within the musical stimuli used in the present study. Most importantly, the effect of valence on memory did not differ between young and older adults. Furthermore, ratings of arousal and valence were clearly dissociated, also for older adults, suggesting there to be independent emotional identification of these two dimensions for the presented music stimuli.

It is worth mentioning that some authors have argued that music evoking negative emotions is very rare (Baumgartner et al., 2006a,b). However, as illustrated by film music, music has succeeded in representing and evoking emotions like sadness, rage, or fear, which lay within the negative valence spectrum (Boltz et al., 1991; Cohen, 2001; Boltz, 2004; Juslin and Laukka, 2004; Mitterschiffthaler et al., 2007; Juslin and Västfjäll, 2008; for a review on music emotions see Kawakami et al., 2014). To this end, the present study contributes toward the differentiation of arousal and valence dimensions on music in an effort to understand their effects on memory. The stimuli used in this study were able to express the intended emotions resulting from the manipulation of arousal and valence dimensions as attested by emotional ratings.

In the present study, the participants were explicitly instructed to remember the musical excerpts while rating emotional valence and arousal of each excerpt during the encoding phase. It is possible that intentional encoding, together with the emotional rating task, may have elicited deeper encoding for all items (Craik and Lockhart, 1972), posing one important limitation to the present study. It could be argued that emotional effects on memory are more prominent for shallow encoding tasks than deep encoding tasks (Ritchey et al., 2011). In contrast, other studies (Hess et al., 2013; Broderick et al., 2014) did not found differences in emotional memory when comparing intentional to incidental encoding instructions. Nevertheless, we cannot rule out that a shallow encoding task may have resulted in greater emotional impact than our intentional encoding/emotional rating task, or even had emphasized differences between age groups.

Another caveat of this study is that although both groups had comparable educational levels, musical expertise, and anxiety levels, our sample was small, with 15 participants in each group, and age was more variable within the old group than the young group. To enhance the sensitivity to age differences, we selected only participants younger than 25 years old and older than 60 years old. A larger sample of participants that may gradually cover all ages may support the generalization of the present findings. Furthermore, studies particularly looking at other psychological factors within a broader aging population may contribute to a better understanding of which conditions could affect emotional memory in aging.

The present findings contribute to the investigation of arousal and valence effects of music on memory in aging. This has important clinical implications, suggesting that the ability to detect emotional valence and arousal in music and recognize emotional music does not seem to be affected by aging, which might explain the efficacy of interventions such as the reminiscence therapy cued by music (Woods et al., 2005; Cotelli et al., 2012). The increasing demands for intervention programs to promote healthy aging and the preservation of cognitive functions, such as memory, can benefit from scientific evidence as such reported here (Reijnders et al., 2013). Based on its emotional power, music has been increasingly used in therapeutic settings with dementia and aging populations (Clément et al., 2012; Narme et al., 2014; Särkämö et al., 2014; for a review see McDermott et al., 2012; Raglio et al., 2012; Ueda et al., 2013). Understanding how emotional characteristics of music may contribute to memory can help to develop interventions and activities that promote healthy aging. In light of these findings, future studies with more detailed sampling of an aging population, eventually including a range from healthy to different degrees of cognitive impairment, and a variety of music genre, may further contribute to explore the potential benefits of music interventions in aging.

## Conclusion

In sum, although older adults performed generally worse than younger adults on music recognition, we were not able to demonstrate age differences on emotional memory for music. The musical excerpts selected for this study allowed the assessment of arousal and valence effects of emotion on recognition, providing equal distinctiveness between the four resulting emotional combinations of arousal and valence. Overall, we present novel evidence on emotional memory for music in young and older adults, immediately and after 24 h delay, by manipulating both arousal and valence dimensions. Immediate recognition results showed that low-arousing but not high-arousing music could be modulated by valence. Both groups showed a benefit for positive as compared to negative music when arousal was low. However, recognition after a 24 h delay, showed a benefit for high over low arousing stimuli and an effect of valence whereby negative excerpts were better recognized than positive excerpts. These findings highlight the impact of emotion on consolidation in memory. Future studies including larger samples of participants covering a broader age range and isolating factors like depth of encoding are highly encouraged to further understand emotional memory for music and how it could be affected by aging.

### Conflict of interest statement

The authors declare that the research was conducted in the absence of any commercial or financial relationships that could be construed as a potential conflict of interest.
