# Supplementary material for: Emotional memory for musical excerpts in young and older adults
Source: Front Aging Neurosci. 2015 Mar 12;7:23. doi: 10.3389/fnagi.2015.00023 (PMC4357296; doi:10.3389/fnagi.2015.00023)
Supplement: Supplementary file 1 [file Table1.DOCX]

| **Table 1.** List of symphonies from which excerpts were extracted | | | | | | | | | | | |
| --- | --- | --- | --- | --- | --- | --- | --- | --- | --- | --- | --- |
|  | **TARGET** |  |  |  | **IMMEDIATE DISTRACTOR** | |  |  | **DELAYED DISTRACTOR** | |  |
| **CATEGORY** | **Composer** | **Symphony** | **Year** |  | **Composer** | **Symphony** | **Year** |  | **Composer** | **Symphony** | **Year** |
| **V+A+** | Charles Villiers Stanford | 1 | 1876 |  | Charles Villiers Stanford | 4 | 1888 |  | Charles Villiers Stanford | 4 | 1888 |
|  | Louise Farrenc | 3 | 1847 |  | Louise Farrenc | 2 | 1845 |  | Louise Farrenc | 2 | 1845 |
|  | Charles Gounod | 1 | 1855 |  | Charles Gounod | 1 | 1855 |  | Charles Gounod | 2 | 1855 |
|  | Bohuslav Martinu | 1 | 1942 |  | Ruperto Chapí | Sinfonía en Re | 1879 |  | Leevi Madetoja | 3 | 1922 |
| **V+A-** | Hans Huber | 1 | 1882 |  | Hans Huber | 3 | 1902 |  | Hans Huber | 3 | 1902 |
|  | George Onslow | 4 | 1833 |  | George Onslow | 3 | 1830 |  | George Onslow | 4 | 1833 |
|  | Peder Gram | 2 | 1925 |  | Peder Gram | 1 | 1914 |  | Peder Gram | 2 | 1925 |
|  | Heinrich von Herzogenberg | 2 | 1889 |  | Wilhem Peterson-Berger | 4 | 1929 |  | William Sterndale Bennett | The Naiads | 1836 |
| **V-A+** | Gsta Nystroem | 3 | 1946 |  | Gsta Nystroem | 3 | 1946 |  | Gsta Nystroem | 1 | 1929 |
|  | Joachim Raff | 6 | 1973 |  | Joachim Raff | 10 | 1879 |  | Joachim Raff | 10 | 1879 |
|  | Louis Glass | 2 | 1899 |  | Louis Glass | 2 | 1899 |  | Louis Glass | 1 | 1894 |
|  | Kurt Atternerg | 1 | 1909 |  | Felix Draeseke | 3 | 1885 |  | Danil de Lange | 1 | 1868 |
| **V-A-** | George Templeton Strong | Die nacht | 1913 |  | George Templeton Strong | Le roi Arthur | 1890 |  | George Templeton Strong | Le roi Arthur | 1890 |
|  | Hugo Alfven | 3 | 1906 |  | Hugo Alfven | 2 | 1899 |  | Hugo Alfven | 2 | 1899 |
|  | Vtzslav Novk | Eternal longing | 1904 |  | Vtzslav Novk | Eternal longing | 1904 |  | Vtzslav Novk | Toman and the woods | 1907 |
|  | Andreas Halln | Gustaf Wasas Saga | 1897 |  | Willem Pijper | Six symphonic epigrams | 1928 |  | Edgar Bainton | 2 | 1939 |
